# Effectiveness of BNT162b2 and ChAdOx1 against SARS-CoV-2 household transmission: a prospective cohort study in England

**DOI:** 10.12688/wellcomeopenres.17995.2

**Published:** 2023-11-02

**Authors:** Samuel Clifford, Pauline Waight, Jada Hackman, Stephane Hué, Charlotte M. Gower, Freja CM Kirsebom, Catriona Skarnes, Louise Letley, Jamie Lopez Bernal, Nick Andrews, Stefan Flasche, Elizabeth Miller

**Affiliations:** 1 Centre for Mathematical Modelling of Infectious Diseases, London School of Hygiene & Tropical Medicine, London, WC1E 7HT, UK; 2 Department of Infectious Disease Epidemiology, London School of Hygiene & Tropical Medicine, London, WC1E 7HT, UK; 3 National Infection Service, UK Health Security Agency, London, NW9 5EQ, UK

**Keywords:** covid, vaccination, secondary attack rate, SARS-CoV-2, household transmission

## Abstract

**Background:**

The ability of SARS-CoV-2 vaccines to protect against infection and onward transmission determines whether immunisation can control global circulation. We estimated the effectiveness of Pfizer-BioNTech mRNA vaccine (BNT162b2) and Oxford AstraZeneca adenovirus vector vaccine (ChAdOx1) vaccines against acquisition and transmission of the Alpha and Delta variants in a prospective household study in England.

**Methods:**

Households were recruited based on adult purported index cases testing positive after reverse transcription-quantitative (RT-q)PCR testing of oral-nasal swabs. Purported index cases and their household contacts took oral-nasal swabs on days 1, 3 and 7 after enrolment and a subset of the PCR-positive swabs underwent genomic sequencing conducted on a subset. We used Bayesian logistic regression to infer vaccine effectiveness against acquisition and transmission, adjusted for age, vaccination history and variant.

**Results:**

Between 2 February 2021 and 10 September 2021, 213 index cases and 312 contacts were followed up. After excluding households lacking genomic proximity (N=2) or with unlikely serial intervals (N=16), 195 households with 278 contacts remained, of whom 113 (41%) became PCR positive. Delta lineages had 1.53 times the risk (95% Credible Interval: 1.04 – 2.20) of transmission than Alpha; contacts older than 18 years old were 1.48 (1.20 – 1.91) and 1.02 (0.93 – 1.16) times more likely to acquire an Alpha or Delta infection than children. Effectiveness of two doses of BNT162b2 against transmission of Delta was 36% (-1%, 66%) and 49% (18%, 73%) for ChAdOx1, similar to their effectiveness for Alpha. Protection against infection with Alpha was higher than for Delta, 69% (9%, 95%) *vs.* 18% (-11%, 59%), respectively, for BNT162b2 and 24% (-41%, 72%) *vs.* 9% (-15%, 42%), respectively, for ChAdOx1.

**Conclusions:**

BNT162b2 and ChAdOx1 reduce transmission of the Delta variant from breakthrough infections in the household setting, although their protection against infection within this setting is low.

## Introduction

The rapid development of safe and effective coronavirus disease 2019 (COVID-19) vaccines using both novel and traditional platforms, is an unprecedented scientific achievement. The United Kingdom was the first country to launch a national COVID-19 vaccination programme with the rollout of the Pfizer-BioNTech mRNA vaccine (BNT162b2) on 8th December 2020, followed shortly after by the Oxford AstraZeneca adenovirus vector vaccine (ChAdOx1). By September 2021, over 40% of the world’s population had received at least one dose of a COVID-19 vaccine, whether an mRNA, adenovirus vector, or inactivated whole virion vaccine ^1^. In most countries, vaccine deployment has been focussed on direct protection of those individuals at the greatest risk of a severe outcome of SARS-CoV-2 infection, including the elderly and those with co-morbidities. Health care workers and others who, if infected, pose a transmission risk to vulnerable individuals, have also been identified as a priority group for vaccination.

The primary outcome of the efficacy trials of the currently authorised COVID-19 vaccines was symptomatic laboratory confirmed SARS-CoV-2 infection, with little information generated on protection against severe COVID-19 infection nor on the ability of the vaccines to prevent onward transmission in those infected. There is now a growing body of evidence from observational studies showing high protection against severe COVID-19 from inactivated whole virion, mRNA, and adenovirus vector vaccines ^2– 4^ but information on protection against transmission is still limited ^5^. Attempts have been made to infer protection against transmission by comparing the viral load in the nasopharynx of vaccinated individuals with breakthrough infections with that in unvaccinated cases, using cycle threshold (Ct) values as a proxy ^6^. Other approaches have used routine diagnostic PCR testing data, constructing households based on individuals’ addresses or identifying them with contact tracing, and to estimate secondary attack rates by vaccination status of the index case. However, these studies are potentially subject to ascertainment bias as they are reliant on the testing behaviour of household contacts ^7– 9^.

Here we report the results of a prospective household transmission study set up by Public Health England (PHE) (now the UK Health Security Agency) in January 2021 to assess the effect of the vaccination history of index cases with COVID-19 on transmission of SARS-CoV-2 to household contacts, and the protection afforded to vaccinated contacts under conditions of household exposure.

## Methods

### Data

***Households.*** The procedures for household recruitment and laboratory testing are the same as those used in the household transmission study conducted prior to vaccine availability and are detailed elsewhere ^10^. In brief, infected index cases, identified *via* community testing in England (known as Pillar 2 testing), and their consenting household contacts are recruited by study nurses, on average, three days after their initial PCR test. No additional measures were taken in the study to prevent household transmission. The vaccination status of index cases and their household contacts is obtained by data linkage with the National Immunisation Management System (NIMS) for England and checked with participants by the study nurse at the time of recruitment. Self-testing kits for the index case and household contacts to take combined nose and throat swabs on Day 1 (day of recruitment), Day 3 and Day 7 are couriered to households and subsequently tested by dual target PCR at PHE Colindale (ORF and E genes). PCR positive swabs are sequenced as part of the COG-UK initiative ^11^. Household contacts were defined as infected if one or more swabs was PCR positive.

The household transmission study is ongoing and inclusion in this analysis is based on participants having returned at least one swab, being either unvaccinated or vaccinated with one or two doses of either BNT162b2 or ChAdOx1 with the vaccination dates recorded in the national vaccination register, and the age at time of recruitment and the date of onset of symptoms (fever, cough, runny nose, sore throat, shortness of breath, loss of taste or smell, nausea, diarrhoea, muscle/body pain, headache or other) recorded.

The analysis code used can be found as *Extended data* ^12^.

### Statistical analysis

All analysis was conducted in R Project for Statistical Computing (RRID:SCR_001905) 4.1.1 ^13^ with Bayesian models fit using the rjags package (RRID:SCR_017573) ^14^. The secondary attack rate (SAR) for each combination of case and contact is estimated here by predicting the probability an unseen contact acquires an infection from an infected case given the vaccination history and age of each and the index case’s variant. As the observed SARs in this study were high, model-estimated odd ratios poorly approximate relative risks. Thus, effect estimates are calculated as risk ratios (RRs) of SARs. Unless mentioned otherwise, the baseline age groups for such comparisons were adult index cases younger than 50 years old and contacts at least 18 years old. The different age grouping for cases and contacts were chosen because of the different age distribution in both groups ( Table 2). The predicted SARs and RRs are summarised with medians and 95% credible intervals.

***Household secondary attack rate.*** We fit a Bayesian hierarchical linear model with Bernoulli likelihood for the probability that a household contact of an index case acquires a SARS-CoV-2 infection within a week of recruitment. The model independently estimates both a protective effect for vaccinated contacts against infection and a reduction in transmission for vaccinated cases. The effect of the first dose is assumed to only occur 21 days after the vaccination is received, and an additional effect of the second dose requires at least seven days have passed since the second vaccination as in the SIREN study, which considers the effectiveness of BNT162b2 in healthcare workers in England ^15^. These effects are assumed to depend on the vaccine product, and number of doses thereof, received by both the index case and the contact ( Table 1). The probability of acquiring infection is also assumed to depend on the age of both the case and contact, and the circulating lineage. Vaccine effectiveness is calculated for both protection against infection and reduction of transmission as 1 -RR for RRs of household SARs with and without the vaccine. For such, the SARs were sampled during the Markov Chain Monte Carlo (MCMC) sampling, for each combination of variant and case and contact vaccine status (1 or 2 doses for each product) and age group, against a baseline of that case-contact pair and variant in the absence of any vaccination.

**Table 1.** Number of contacts with listed vaccine status for each case vaccine status (N). Numbers in brackets show the additional individuals included in the sensitivity analysis (n). BNT162b2, Pfizer-BioNTech mRNA vaccine; ChAdOx1, Oxford AstraZeneca adenovirus vector vaccine.

Case	1 ChAdOx1 N(n)	2 ChAdOx1 N(n)	1 BNT162b2 N(n)	2 BNT162b2 N(n)	None N(n)
1 ChAdOx1	17 (4)	1	3	4	23 (5)
2 ChAdOx1	2 (1)	26 (5)	7 (1)	12 (1)	21 (9)
1 BNT162b2	6	1	15 (2)	2	33 (2)
2 BNT162b2	9	8	4	10	9
None	6	2	4	5	48 (2)

For the model of secondary attack rate, the likelihood is


$$\begin{array}{l} \;y_{i}\;\sim \;\text{Bern}(p_{i})\\ \log\left(\frac{p_{i}}{1-p_{i}}\right)\;=\;\beta_{0}+\delta_{V_{i}}+\\ \;\beta_{1,v_{i},V_{i}}I\left(d_{1,i,\text{contact}}\geq k_{1}\right)+\beta_{2,v_{i},V_{i}}I\left(d_{2,i,\text{contact}}\geq k_{2}\right)+\\ \;\gamma_{1,v_{i},V_{i}}I\left(d_{1,i,\text{case}}\geq k_{1}\right)+\gamma_{2,v_{i},V_{i}}I\left(d_{2,i,\text{case}}\geq k_{2}\right)+\\ \;\varepsilon_{\text{contact}}I\left(A_{i,\text{contact}}<a_{\text{contact}}\right)+\varepsilon_{\text{case}}I\left(A_{i,\text{case}}\geq a_{\text{case}}\right) \end{array}$$

where **I** is an indicator function, which is 1 when its input is true and 0 otherwise, and *d _j,i,c_* is the number of days since the *j*th dose of vaccine product *υ* was given to either contact *i* or their household index case (indexed by *c*) who is infected with variant *V _i_* A fixed effect, *δ*, accounts for the increased infectivity of Delta beyond that of Alpha. Protection afforded by dose *j* is assumed to begin after *k* = {21,7} days. These are currently fixed, but a distribution may be used instead if there is some observed variability we wish to include. Age effects, *ε*, are assumed to be non-zero when the contact is younger than *A*_*i*,contact_ = 18 and when the case is at least as old as *A*_*i*,case_ = 50. Where *V _i_* was missing due to that household’s swabs not being sequenced, it was sampled at each step of the MCMC from a Bernoulli distribution with its single parameter representing the modelled proportion of sequenced Pillar 2 swabs with Delta lineage at time of that household’s Day 1 swabs.

The priors for the model parameters associated with transmission reduction are parameterised as weakly informative normal distributions (with means and precision ( *τ*= *σ* ^–2^))


$$\begin{array}{c} \gamma_{j,v,V}∼(\gamma_{j,0,V},\tau_{\gamma })\\ \gamma_{j,0,V}∼(0,10^{6})\\ \tau_{\gamma }=\sigma_{\gamma }^{-2}\\ \sigma_{\gamma }∼\text{Exp}(0.3) \end{array}$$

We included penalised complexity priors to favour the NULL model of no protective vaccine effect against transmission. The penalised complexity prior on the standard deviation implies a prior probability of it exceeding 10 of 0.05.

For the infection protection parameters, informative priors are derived from reported vaccine efficacy of the two vaccine products against the Alpha (B.1.1.7) and Delta (B.1.617.2) variants of SARS-CoV-2 ^16^. The priors are normally distributed for the log-odds ratios, with mean and precision parameters,


$$\begin{array}{l} \beta_{1,1,A}∼(\log\;0.5,2)\\ \beta_{1,2,A}∼(\log\;0.43,0.25)\\ \beta_{2,1,A}∼\left(\log\frac{0.25}{0.5},2\right)\\ \beta_{2,2,A}∼\left(\log\frac{0.06}{0.43},0.25\right) \end{array}\;\begin{array}{l} \beta_{1,1,D}∼(\log\;0.65,2)\\ \beta_{1,2,D}∼(\log\;0.67,0.25)\\ \beta_{2,1,D}∼\left(\log\frac{0.33}{0.65},2\right)\\ \beta_{2,2,D}∼\left(\log\frac{0.12}{0.67},0.25\right) \end{array}$$

Here we have taken the point estimates of the log odds ratios and doubled the standard errors, rounding to the nearest 0.5, in order to provide informative priors with additional variance that ensure that the posteriors are still sensitive to the data. As the *β*_2 *,υ,V*_ represent the marginal effect of the second dose, we derive *B*_2 *,υ,V*_ = *β*_1 *,υ,V*_+ *β*_2 *υ,V*_, the log-odds of the effect of double vaccination against variant *V* with vaccine product *υ*.

The effects of age have informative priors derived from Davies *et al.* ^17^, for under-18s acquiring infection, *ε*_contact_~ *N*(log⁡0.50,24), and similar to post-hoc calculations in Goldstein *et al.* ^18^ based on Yousaf *et al.* ^19^ for relative transmissibility of a >50y old case, *ε*_case_~ *N*(log⁡1.86,4.67).

To determine how informative the priors above are, we replace the informative priors above for all *β _j,υ,V_* with a weakly informative *N*(0, $\sigma_{\beta }^{-2}$ ) prior with *σ _β_* ~Exp(0.3) and the effects of age each having a weakly informative *N*(0,10 ^–6^) prior.

***Lineage.*** At the start of data collection, the B.1.1.7 (Alpha) SARS-CoV-2 variant was most prevalent in the United Kingdom, and an increasing proportion of swabs sequenced by Pillar 2 testing were identified as B.1.617.2 (Delta) variant over time ^20^. Where sequencing was not available to determine the variant for a positive swab, the probability that it was the Delta variant was estimated from the date of sampling and a logistic regression model fit to the number of weekly cases identified through Pillar 2 that were either Alpha or Delta variant.

***Participants’ age.*** Vaccine eligibility and type is correlated with age and date of vaccination. This is because from 7th April 2021 the BNT162b2 vaccine was recommended for individuals under 30 years old in preference to ChAdOx1, which was then extended to those between 30 and 40 years old from 7th May 2021 ^21^ and also because, apart from those in high risk groups, vaccination was not offered to the general 16–17 year old population until August 2021 ^22^ and the general 12–15 year old population until September 2021 ^23^. We account for age in the model by considering that children under 18 years old will have decreased susceptibility to infection, compared to adults ^17^, and that older adults are more likely to transmit ^18^. While the study did not specifically recruit only adult index cases, the minimum age of index cases was 21 years old. The median age of index cases was 48 years old and so we split adults into younger (between 18 and 49 years old) and older (at least 50 years old) age groups. Few participants were older than 65 years old, so we did not distinguish between groups aged 50–64 and 65+ years old. We did not adjust for prior infection status as information on this was incomplete at the time of data lock, nor for gender as this was previously shown not to be a factor in determining household transmission ^10^. Table 2 shows the age and vaccine status breakdown of index cases and their household contacts.

**Table 2.** Number of index cases and their household contacts with listed vaccine status for each age group (N). Numbers in brackets show the additional individuals included in the sensitivity analysis (n). There are no index cases younger than 18 years old. BNT162b2, Pfizer-BioNTech mRNA vaccine; ChAdOx1, Oxford AstraZeneca adenovirus vector vaccine.

Status	Vaccine	Age group			
		<18 N(n)	18-49 N(n)	50-64 N(n)	65+ N(n)
Case	1 ChAdOx1	0	15 (2)	17 (2)	3 (1)
	2 ChAdOx1	0	20 (3)	26 (4)	1
	1 BNT162b2	0	22 (1)	13 (2)	2
	2 BNT162b2	0	13	17	1
	None	0	33	12 (1)	0
Contact	1 ChAdOx1	0 (1)	13 (2)	22 (2)	5
	2 ChAdOx1	0	13 (2)	22 (2)	3 (1)
	1 BNT162b2	0	22 (2)	9	2 (1)
	2 BNT162b2	0	14 (1)	16	3
	None	67 (7)	55 (8)	11 (2)	1 (1)

***Infection history dynamics.*** PCR positivity relative to the onset of symptoms was estimated using data from all symptomatic cases and contacts, with pseudo-absences generated to simulate the time of infecting exposure. Comparison is made for each combination of vaccine product, number of doses, and variant against the corresponding unvaccinated group.

***Identification of non-household transmission.*** As per the study design, the index case for each household was by default considered to be the individual who presented for Pillar 2 testing. To reduce the risk of misclassification bias we excluded from the analyses all households where both the index case and an infected household contact were symptomatic and the index case’s symptoms appeared more than two days after the contact’s symptoms.

To further reduce the potential for misclassification bias, a phylogenetic approach was used to identify apparent secondary cases in the household who were in fact infected elsewhere. If none of the sequences from a contact clustered with at least one of the sequences from the household’s index case, then this was considered as evidence for an infection acquired outside of the household; therefore, the contact was excluded from the downstream analysis.

Whole-genome Illumina reads were retrieved from the European Nucleotide Archive (ENA) (RRID:SCR_006515) under the accession PRJEB37886. Consensus genomes were generated using the Snippy pipeline mapping to the reference genome NC_045512.2 ^24^. Highly ambiguous and/or homoplastic sites were masked in the consensus alignment as described by de Maio *et al.* ^25^. A maximum-likelihood phylogeny was reconstructed from the consensus genomes under the Hasegawa-Kishino-Yano (HKY) model of nucleotide substitution with 1,000 ultrafast bootstrap replicates to assess branch supports and visualized in iTOL (RRID:SCR_018174) ^26, 27^.

ClusterPicker was used to identify clusters of transmission in the phylogeny ^28^. These were defined as clusters of sequences with patristic distances of no more than 2 SNP (6.6 × 10 ^-5^ substitutions/site) ^29^ and bootstrap support of at least 70%.

## Results

By September 10th, 2021, a total of 213 index cases and 312 contacts had been recruited and met the criteria for inclusion at that time. Two contacts were removed due to lack of genomic proximity (outlined below), which resulted in the removal of each of their households as there were no further contacts. The serial interval was two (95% range: -6, 10) days. A total of 16 households with their respective index cases and a total of 32 contacts were excluded from the main analysis because at least one infected household contact presented symptoms more than two days before the index case. Thus, the main analysis was performed on 195 index cases and their 278 contacts. Households had between one and seven contacts, with a mean of 2.2, median of 2, and standard deviation of 1.2. The mode number of household contacts was 1.

Of the included individuals, 175 index cases (90%) and 113 (41%) contacts tested positive for SARS-CoV-2 at least once in the week since recruitment. For the identification of non-household transmission, sequencing information was available for 121 (69%) and 80 (71%) of those, respectively.

 A total of 24% of contacts were less than 18 years old, and therefore not eligible for vaccination at the time. The proportion of at least partially vaccinated (adult) household index cases and contacts was 77% and 69%, respectively ( Table 2). Only 10 index cases (5%) were asymptomatic, reflecting the bias of Pillar 2 testing in the UK towards detecting mostly symptomatic infections. Fully vaccinated cases had received their second dose on average 70 days before enrolment and fully vaccinated contacts 71 days.

### Prevalence of lineages

For the main analyses, of the 195 index cases analysed here, 99 were identified as infected with B.1.1.7 (Alpha), 24 with B.1.617.2 (Delta), 20 did not test positive again after recruitment, and 52 were of unknown lineage as their PCR-positive swabs had not yet been sequenced. Of the 72 individuals without information on the infecting lineage, we estimated that 18 were likely of Alpha and 54 were likely of Delta lineage based on the date of sampling and the national prevalence of lineages at the time. That is, 60% of index cases had an Alpha variant infection and the remainder were Delta.

### Identification of non-household transmission

Sequencing information for both index case and contact were available for 92 PCR positive case-contact pairs across 79 households. In total, 345 whole-genome sequences (including longitudinal samples) were available for analyses, a majority of which were of Alpha variant (82.6%) and the remainder were Delta (17.4%).

**Figure 1.**
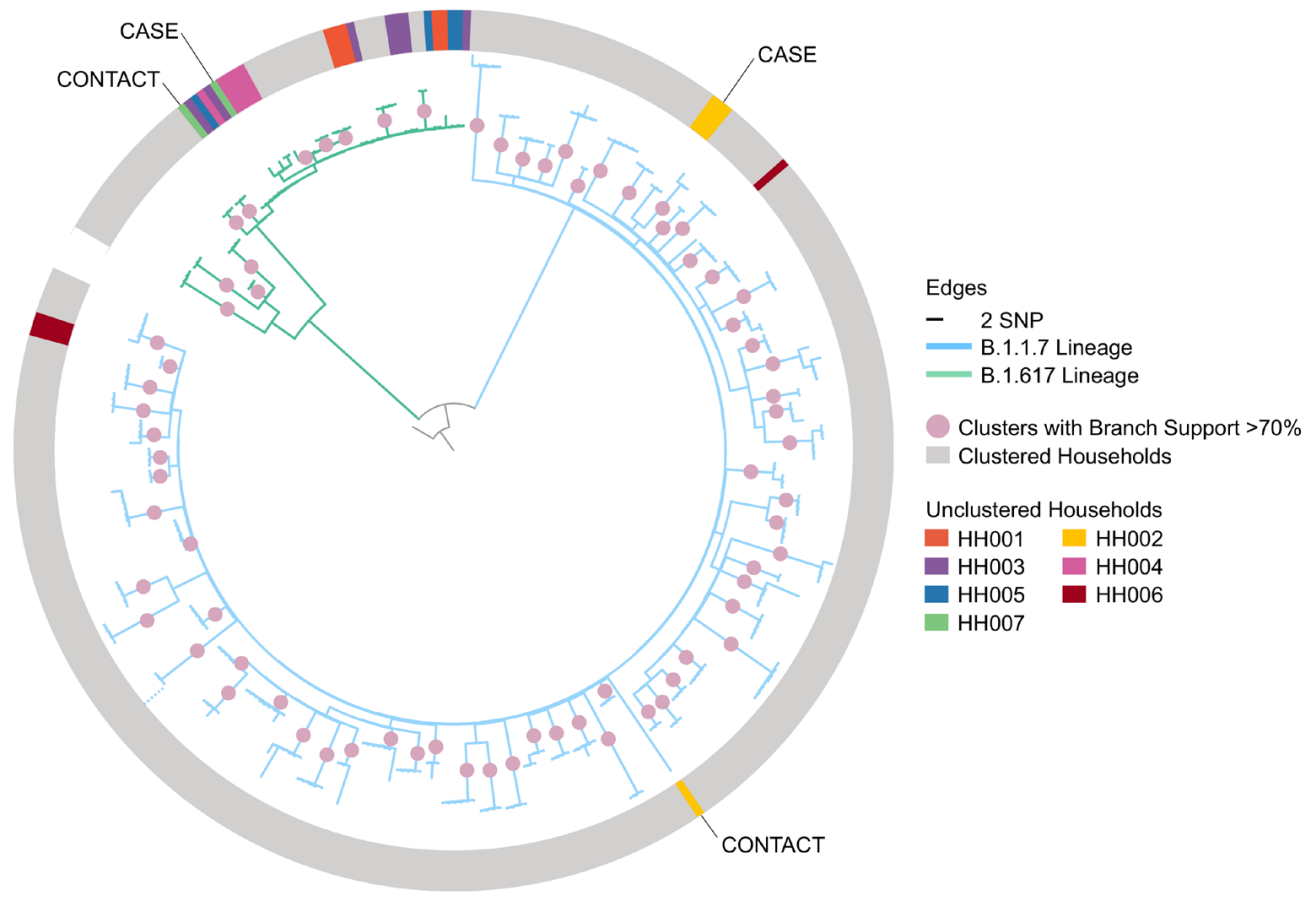
Maximum-likelihood phylogeny of household index cases and contacts’ sequences with 1,000 ultrafast bootstrap replicates rooted to the reference sequence with a scaled bar of 2 SNP (6.6 × 10 ^-5^ substitutions/site). The dotted line at bottom left indicates where a single long branch was collapsed for visualisation. The non-grey shading on the outer ring represents non-clustered households where sequences are coloured by their households. HH002 and HH007 were the only households where none of the contacts’ sequences clustered with that of their household’s index case and this is evidence that the contact could have acquired the infection elsewhere and is thus excluded from the analysis. SNP, Single Nucleotide Polymorphism.

The phylogeny provided evidence that in two households the contact of the recruited index case had acquired infection elsewhere ( Figure 1, households HH002 and HH007). Five households that did not form unique clusters in the phylogeny did not meet the exclusion criteria: in two a sequence from an index case did not cluster with the remaining household sequences but another sequence from the same index case did (HH004 and HH006), while the other three households did not have sufficient bootstrap support to be a part of a cluster (HH001, HH003, and HH005). Of the remaining households, 72 (91%), formed unique, household-specific clusters that included all and only sequences of members of the household, indicating likely direct transmission within the household.

### Age and lineage effects

We estimate that in the absence of vaccination of either case or contacts, Delta lineage infections were much more transmissible within the household than Alpha lineage infections (RR: 1.53, 95% Credible Interval: 1.04, 2.20 for adult cases <50 years old). Children younger than 18 years old were less likely than adults to acquire an Alpha infection (RR: 0.67, 95%: 0.52, 0.83) and just as likely to acquire a Delta infection (RR: 0.98, 95%: 0.86, 1.07). Compared to a baseline of index cases aged between 18 and 49 years old, those 50 years old and over did not transmit either an Alpha (RR: 1.15, 95%: 0.89, 1.47) or Delta (1.08, 95%: 0.96, 1.30) infection to any greater degree at a 95% level of credibility.

### Effectiveness of vaccination

Either one or two doses of BNT162b2 provide contacts with a protective effect against infection from a symptomatic index case with Alpha variant SARS-CoV-2 with a vaccine effectiveness of 51%, (95% credible interval: 4%, 83%) and 69% (95% credible interval: 9%, 95%), respectively ( Table 3). At 0% (-33%, 39%) and 18% (-11%, 59%) the effectiveness of one and two doses of BNT162b2 against infection with the Delta variant was lower than against Alpha. The protection offered by ChAdOx1 to either variant after two doses was also low, with effectiveness against Alpha of 24% (-41%, 72%) and against Delta of 9% (-15%, 42%).

**Table 3.** Median VE and 95% credible intervals for infection protection in contacts and transmission reduction in cases, by variant, vaccine product, and number of doses. VE, vaccine effectiveness; BNT162b2, Pfizer-BioNTech mRNA vaccine; ChAdOx1, Oxford AstraZeneca adenovirus vector vaccine.

Variant	Vaccine	Doses	VE infection	VE transmission
Alpha	ChAdOx1	1	-1% (-42%, 36%)	-9% (-63%, 28%)
		2	24% (-41%, 72%)	36% (-29%, 74%)
	BNT162b2	1	51% (4%, 83%)	23% (-18%, 54%)
		2	69% (9%, 95%)	57% (2%, 85%)
Delta	ChAdOx1	1	-1% (-28%, 28%)	15% (-17%, 58%)
		2	9% (-15%, 42%)	49% (18%, 73%)
	BNT162b2	1	0% (-33%, 39%)	10% (-20%, 54%)
		2	18% (-11%, 59%)	36% (-1%, 66%)

We estimate that the effectiveness of one and two doses of BNT162b2 against onward transmission from cases infected with the Alpha variant was 23% (-18%, 54%) and 57% (2%, 85%), respectively, and for Delta variant one and two doses reduce transmission by 10% (-20%, 54%) and 36% (-1%, 66%), respectively. RRs for the protective effect of BNT162b2 over ChAdOx1 for one and two doses of against both Alpha and Delta variants indicate that at 95% credibility there is no evidence for a difference between the effectiveness of the two vaccine products. Specifically, the RRs for acquisition of Alpha and Delta after two doses of BNT162b2 *vs.* ChAdOx1 for an adult contact are 0.41 (0.06, 1.70) and 0.92 (0.52, 1.31), respectively.

### Secondary attack rates

**Figure 2.**
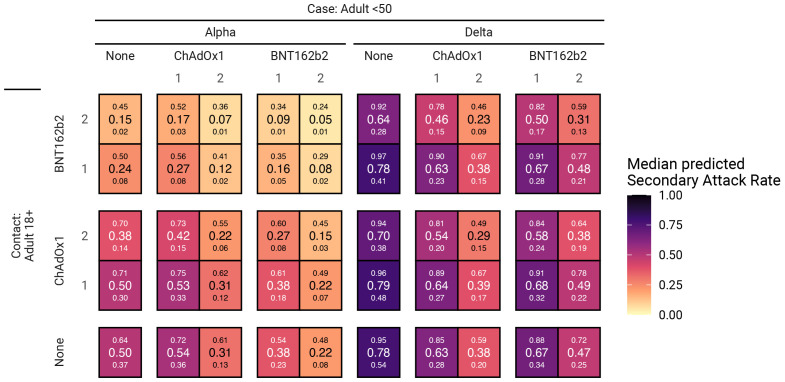
Predicted SARs for each combination of vaccine status of case and contact. Large numbers inside cells are the median SAR of the posterior distribution, with the small numbers below and above corresponding to the 95% credible interval. SAR, secondary attack rate; BNT162b2, Pfizer-BioNTech mRNA vaccine; ChAdOx1, Oxford AstraZeneca adenovirus vector vaccine.

The estimated secondary household attack rate among adults in an unvaccinated household was 50% (37%, 64%) for the Alpha variant and 78% (54%, 95%) for the Delta variant ( Figure 2).

BNT162b2 is very effective against Alpha variant infection when either the case or contact are vaccinated, and especially when both have received two doses ( Figure 2). SARs for Delta variant infection in unvaccinated case-contact pairs are substantially higher. Full (two dose) vaccination with either vaccine is still effective against Delta infection when both the case and contact are vaccinated, at least halving the SAR; *e.g.*, case and contact both fully vaccinated with BNT162b2 has an SAR of 31% (13%, 59%). Notably, the reduced susceptibility to infection of (unvaccinated) individuals under 18 years old results in Alpha SARs that are no greater than those seen in adult contacts who have received two doses of ChAdOx1. Conversely, for Delta infections, there is no reduced susceptibility for those aged under 18 years and so unvaccinated under- and over-18s have similar probability of becoming infected, with a single dose of either vaccine providing no discernible protection.

### Sensitivity analysis

Sensitivity analysis was conducted by including the 16-index case-contact pairs with serial intervals less than two days. This did not qualitatively change our results. The absence of informative priors on the protective vaccine effects against infection led some of the vaccine effectiveness against infection in our study to be re-attributed to effectiveness against onward transmission or to age effects.

### Infection history dynamics

We estimate that within a week of symptom onset, the relative risk of symptomatic cases testing PCR positive is near identical for vaccinated and unvaccinated participants. For cases infected with the Alpha variant, there was little difference in PCR positivity generally between vaccinated and unvaccinated cases, while in cases infected with the Delta variant the proportion of participants with PCR detectable infection in participants fully vaccinated with BNT162b2 declined about four days before that in unvaccinated participants. In participants fully vaccinated with ChAdOx1 the effect was slightly less pronounced with PCR positivity declining two to three days earlier in vaccinees than in unvaccinated.

## Discussion

In this prospective household-based study of SARS-CoV-2 infection, we showed that both the ChAdOx1 and BNT162b2 vaccines are effective in reducing transmission of the Alpha and Delta variants from those who develop breakthrough infections despite having received two doses. The estimated vaccine effectiveness against acquisition of a Delta infection in the household setting was however low; 9% (-15%, 42%) and 18% (-11%, 59%) after two doses of ChAdOx1 and BNT162b2, respectively. This is much lower than that estimated from cases presenting for Pillar 2 testing in the community for which the effectiveness of two doses of ChAdOx1 against symptomatic infection is estimated as 67.0% (61.3%, 71.8%) and 88.0% (85.3%, 90.1%) for BNT162b2 ^15^. Effectiveness against acquisition of an Alpha infection in the household was substantially higher in our study than that against Delta but still lower than that estimated from Pillar 2 community testing. The lower protection against acquisition in the household likely reflects the prolonged and intense exposure that occurs in this setting. Similarly, although the effectiveness estimates against Delta transmission within the household were moderate at 49% (18%, 73%) and 36% (-1%, 66%) after two doses of ChadOx1 and BNT16b2, respectively, the protective effect in those with breakthrough infections may be higher in the community where exposure is less intense and of shorter duration. The reduction in duration of PCR positivity in breakthrough infections (average of four days shorter for the Delta variant for those infected after two doses of BNT162b2 and around two to three days for ChAdOx1) will also have more of an impact in the community than in the household setting where generation times between infections are short – around 3.5 days for the Delta variant ^30^. Our household contacts were actively followed up with repeated swabbing and showed the high secondary attack rates that occur in this setting; 81% for Delta infections in unvaccinated households but that reduced to 25–40% in households where both index case and contacts were fully vaccinated.

### Comparison with other studies

Our finding of a moderate level of protection against onward transmission from fully vaccinated individuals, with either vaccine and against either variant, is in apparent contrast to a study that similarly followed up contacts reported by the UK test and trace system prospectively, about 90% of whom were in the same household as the index case ^31^. The study estimated a moderate effect of vaccination against infection but no difference in secondary attack rates with the delta variant between fully vaccinated and unvaccinated index cases (24% and 23%, respectively). However, such estimates were neither controlled for age nor vaccination status of the contact. Notably, only four out of 17 (24%) unvaccinated contacts were infected by fully vaccinated index cases, whereas eight out of 20 (40%) unvaccinated contacts were infected by unvaccinated index cases; a reduction in transmission of 41% albeit based on very small numbers. In a similar study from Singapore Delta-exposed fully vaccinated household, contacts were 56.4% less likely to test positive on quarantine exit screening ^32^. Also, this study had insufficient power to detect a significant vaccine effect in onward transmission; the odds of a positive exit screening test for Delta-exposed household contacts was 27% (95% CI: -40, 62) lower in contacts of fully vaccinated index cases. Secondary attack rates in our study were much higher, potentially owing to the regular testing during quarantine and the absence of measures to prevent transmission in the household. This has helped through providing statistical power to the point estimates of the other two studies and show a protective effect against onward transmission. Vaccine effectiveness against onward transmission of 40–80% has been suggested by several retrospective observational studies using either information on the household structure ^7^ or contact tracing ^8, 9^ in combination with routine national COVID-19 notification systems to estimate reductions in secondary attack rates from breakthrough infections. While observational studies are prone to biases introduced by testing behaviour particularly for mild disease manifestations, our study combines prospectively collected longitudinal data from recruited households with a robust analytical framework to confirm that both vaccines reduce transmissibility of breakthrough infections in fully vaccinated individuals.

**Figure 3.**
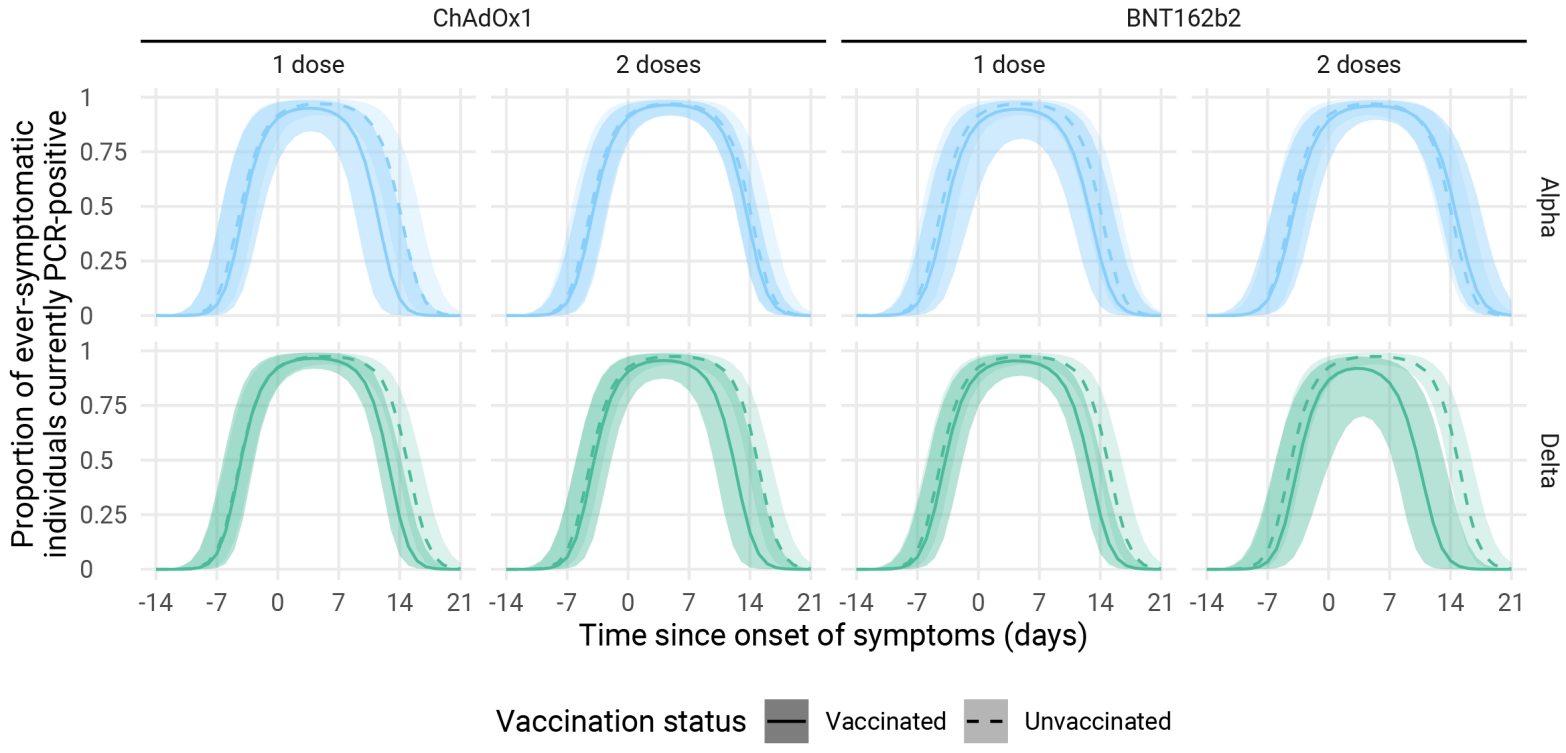
PCR positivity by variant and vaccination status for symptomatic infections (index cases recruited from Pillar 2 testing and the symptomatic household contacts they infected). Lines represent median trajectories, and the ribbon is the 95% credible interval. BNT162b2, Pfizer-BioNTech mRNA vaccine; ChAdOx1, Oxford AstraZeneca adenovirus vector vaccine.

Among symptomatic index cases and contacts, we found a lower rate of PCR positivity within two weeks of symptom onset in all vaccinated groups ( Figure 3). PCR positivity for Delta declined fastest (four days ahead of unvaccinated) in individuals fully vaccinated with BNT162b2. These results largely mirror those in other studies that found enhanced clearance following vaccination ^31^, but raise the question whether enhanced clearance can be the driving mechanism for reduced transmission in a frequent contact household setting. Another mechanism may be that while positivity with the highly sensitive PCR test is similar to that in the unvaccinated, vaccination can reduce ^33^ both peak viral load ^6, 34^ and viral shedding ^35^ although such effects have not been reported in all studies and may be masked by age effects.

### Strengths and limitations of this study

Our study comes with limitations. To minimise the potential for misclassification we restricted the main analyses to only those putative transmission pairs where there was no evidence against direct transmission based on phylogenetic distance (which was available for 63% of all putative transmission pairs) and where symptom onset in the contact did not pre-date that of the index case by more than two days. Our model does not account for the introduction of SARS-CoV-2 into the household from two index cases who acquired the infection separately, opting instead to exclude the most recently PCR-positive index case. Importantly, if there is residual misclassification between infector and infected this would attribute infection protection to transmission protection and *vice versa*.

Only households with adult index cases were recruited into the study. During the time of data collection, children were ineligible for vaccination and so there would be no vaccine effectiveness for reducing transmission to estimate even if such data were included. The inclusion of households with child index cases would, however, provide useful further information on the protection against acquisition for both adult and child contacts of unvaccinated cases, particularly in understanding the risk of children who acquire infection in their school environment and may transmit to family members.

Prior infection status is not included in the model as the data were not available at the time of data lock. This can be incorporated in the model under the same structure as a vaccine product, though this may be difficult when considering protection against a specific variant provided by infection with previously seen variants when the prior infection’s variant is unidentifiable.

The ability to detect an infection in contacts relies on the sensitivity of PCR and the timing of swabs. A vaccinated contact who acquires infection may be less detectable due to a reduction in viral load and/or shorter shedding period ^35^ and may have been detectable between the swabs on days three and seven.

We did not include waning of vaccine protection in our analyses. In the analysed dataset the longest reported time since vaccine receipt was 169 days. While some individuals in the analysis have since become eligible for a booster vaccination over concerns of waning protection, some of this potential effect will have been absorbed in our model in the age structuring because of the strong correlation between age and timing of vaccine eligibility as per the vaccine roll-out strategy in the UK. Lastly, data collection spanned a period of multiple months during which Delta became the dominant strain in circulation in the UK and included participants vaccinated with two different vaccine products; thus, requiring sub-strata analyses and reducing the effective sample size for each strata. We used a Bayesian model that allowed the borrowing of strength through the model hierarchy, and priors allowing us to make use of the heterogeneity in risk factors and not only estimate vaccine effectiveness against transmission in these strata but simultaneously estimate the difference in transmissibility in Alpha and Delta variants and the effectiveness of partially completed dosing schedules. The use of informative priors was integral to disentangling the confounded age and vaccine history effects, which arose due to vaccine product prioritisation and were exacerbated by low counts for case-contact vaccine history combinations. Additionally, we assume that carriage of multiple variants does not occur, with genomic sequencing only showing a single variant.

## Conclusions

Our findings provide robust evidence that vaccination with either BNT162b2 or ChAdOx1 can help to substantially reduce, but not completely prevent, household transmission with SARS-CoV-2. This highlights the importance of vaccines to limit circulation of SARS-CoV-2 particularly in close and prolonged contact indoor settings. The effectiveness of booster doses to further enhance protection against transmission will need to be evaluated to better understand the extent to which we can rely on vaccination for the control of SARS-CoV-2 infection, particularly during winter seasons when most contacts occur in households or household-like settings.

## Ethics approval

UKHSA Research Ethics and Governance Group Statement: Surveillance of COVID-19 testing and vaccination is undertaken under Regulation 3 of The Health Service (Control of Patient Information) Regulations 2002 to collect confidential patient information ( http://www.legislation.gov.uk/uksi/2002/1438/regulation/3/made) under Sections 3(1) (a) to (c), 3(1)(d) (i) and (ii) and 3(3). The study protocol was subject to an internal review by the PHE Research Ethics and Governance Group and was found to be fully compliant with all regulatory requirements. As no regulatory issues were identified, and ethical review is not a requirement for this type of work, it was decided that a full ethical review would not be necessary. All necessary patient/participant consent has been obtained and the appropriate institutional forms have been archived. Oral informed consent for sampling and follow up was obtained by the nurses from household members who were free to decline to participate in the surveillance at any time. Consent for children was obtained by a parent or legal guardian. Only anonymised data were provided to non-UKHSA authors.

## Data Availability

The data necessary to replicate results are available from the authors on request, subject to a data sharing agreement. Requests for the underlying data should be made via the UKHSA office for data release: https://www.gov.uk/government/publications/accessing-ukhsa-protected-data. Analysis code available from: https://doi.org/10.5281/zenodo.7618847 ^12^ License: MIT
