# Supplementary material for: Effectiveness of BNT162b2 and ChAdOx1 against SARS-CoV-2 household transmission: a prospective cohort study in England
Source: Wellcome Open Res. 2023 Nov 2;8:96. Originally published 2023 Feb 22. [Version 2] doi: 10.12688/wellcomeopenres.17995.2 (PMC10697107; doi:10.12688/wellcomeopenres.17995.2)
Supplement: Peer review history

# Reviewer report: Reviewer response for version 2

**DOI:** 10.21956/wellcomeopenres.22475.r69545

**Published:** 2023-11-23

**Authors:** Tim K Tsang

**Affiliations:** 1 The University of Hong Kong, Hong Kong Special Administrative Region, China

I am ok with the response.

Is the work clearly and accurately presented and does it cite the current literature?

Partly

If applicable, is the statistical analysis and its interpretation appropriate?

Partly

Are all the source data underlying the results available to ensure full reproducibility?

Partly

Is the study design appropriate and is the work technically sound?

Yes

Are the conclusions drawn adequately supported by the results?

Partly

Are sufficient details of methods and analysis provided to allow replication by others?

Partly

Reviewer Expertise:

biostatistics, epidemiology, infectious disease modelling

I confirm that I have read this submission and believe that I have an appropriate level of expertise to confirm that it is of an acceptable scientific standard.

# Reviewer report: Reviewer response for version 2

**DOI:** 10.21956/wellcomeopenres.22475.r69546

**Published:** 2023-11-16

**Authors:** John Kubale

**Affiliations:** 1 University of Michigan, Ann Arbor, USA

I appreciate the authors thoughtful responses to my comments (including those I made only because I missed where they had already provided the requested information). I have no further concerns with the manuscript and congratulate the authors on this nice addition to the literature.

Is the work clearly and accurately presented and does it cite the current literature?

Yes

If applicable, is the statistical analysis and its interpretation appropriate?

Yes

Are all the source data underlying the results available to ensure full reproducibility?

Yes

Is the study design appropriate and is the work technically sound?

Yes

Are the conclusions drawn adequately supported by the results?

Yes

Are sufficient details of methods and analysis provided to allow replication by others?

Partly

Reviewer Expertise:

Infectious disease epidemiology

I confirm that I have read this submission and believe that I have an appropriate level of expertise to confirm that it is of an acceptable scientific standard.

# Reviewer report: Reviewer response for version 1

**DOI:** 10.21956/wellcomeopenres.19952.r60461

**Published:** 2023-07-17

**Authors:** Tim K Tsang

**Affiliations:** 1 The University of Hong Kong, Hong Kong Special Administrative Region, China

This paper reports the transmission dynamics and VE estimates from a household transmission study. Overall, it is informative and provides insight to the literature. Analysis approach could be improved to make things more accurate. The interpretations of some results could be improved to make it more clear on the meaning of findings.

 Major comments:

1. The Bayesian model in the paper already assumed that contacts could be infected from index only, which may not be accurate. There are many models that can handle this, such as Cauchemez *et al.* ^1^. Optimally such models should be used, then the factors affecting transmission and factors affecting susceptibility could be estimated. At least, it should be mentioned in the limitations.
2. Following 1, the factors estimated by the model could not be interpreted as factor impacting transmissions, since the model is about infection status of contacts. So terms like ‘reducing transmissions’ are not accurate.
3. Following 2, I cannot follow how the model can estimate the VE against transmission. Are you putting the index cases’ vaccination status as a factor and consider this as VE against transmission? If so, these estimates are based on the assumption that contacts could only be infected from index and state this limitation clearly.
4. The force of infection could outside household was not also addressed, and it is again an limitation that should be stated. Some works were done like excluding contacts cases with long serial intervals, but we cannot rule out that two cases with short serial intervals could be both infected from outside household and not household transmission.

Minor comments:

1.  Figure 2. Regarding the median of SAR, do you mean the median of posterior distribution? or for each household, you compute the SAR and then take median? I think it is the former, but please clarify.

Is the work clearly and accurately presented and does it cite the current literature?

Partly

If applicable, is the statistical analysis and its interpretation appropriate?

Partly

Are all the source data underlying the results available to ensure full reproducibility?

Partly

Is the study design appropriate and is the work technically sound?

Yes

Are the conclusions drawn adequately supported by the results?

Partly

Are sufficient details of methods and analysis provided to allow replication by others?

Partly

Reviewer Expertise:

biostatistics, epidemiology, infectious disease modelling

I confirm that I have read this submission and believe that I have an appropriate level of expertise to confirm that it is of an acceptable scientific standard, however I have significant reservations, as outlined above.

## References

[1] : A Bayesian MCMC approach to study transmission of influenza: application to household longitudinal data. *Stat Med* .2004;23(22) : 10.1002/sim.1912 3469-87 10.1002/sim.1912 15505892

# Response

**Published:** 2023-10-19

**Authors:** Stefan Flasche

**Affiliations:** LSHTM, UK

Thanks you for the review. We have addressed the minor comment. You are right to highlight that the inferrence of direction of transmission and accounting for potential transmission from the outside as two key aspects of the sutdy. In household studies like this one, one needs to infer the direction of transmission and to account for the potential for infection from outside the household based on suboptimal information. To limit the risk for the latter we conducted a genomic analysis to identify those secondary cases who would not genomically cluster with the other cases in the household. To address the former, one could, like other have done before, infer the direction of transmission purely based on the relative timing of reported symptoms. However, in this study many HH members were children with mild symptoms that may or may not be related to covid and thus such approach may result in misclassification. Instead we decided to assume that the first case severe enough to seek official testing would be the index case but excluded all households where the dates of symptom onset suggested the index case was unlikely the first HH case. We discussed these issues in the first paragraph of the section on strength and limitations. I hope this is clearer now.

# Reviewer report: Reviewer response for version 1

**DOI:** 10.21956/wellcomeopenres.19952.r57835

**Published:** 2023-06-19

**Authors:** John Kubale

**Affiliations:** 1 University of Michigan, Ann Arbor, USA

Overall, this is a nice paper from Clifford *et al.* that will be a positive addition to the literature. My comments are all fairly minor and are primarily aimed at making the methods employed clearer and more accessible to a broader audience.

- Why the difference in age groups for case and contact comparisons?
- I’m not sure about the independence assumption between protective effect of vaccinated contacts and reduced transmission of vaccinated cases. Wouldn’t there be some clustering between similar groups?
- Should consider adding additional detail on complexity prior. It would also be helpful for a sentence or two on why precision is used here for those less familiar with its benefits for Bayesian modeling.
- Would recommend changing by a factor of 2 to something more intuitive like doubling or 2x causes unnecessary confusion for folks who don’t frequently use this terminology.
- Should clearly state which age group the case transmission prior is for.
- Not clear where in the paper by Yousaf *et al.,* the estimate comes from?
- Negative serial interval?
- 90% of index cases tested positive at least once in the week prior to recruitment, what about the other 10%?
- Based on how index cases were defined/identified they must have tested positive to be recruited, what range did you see in this group in days between positive test and recruitment?
- In one paragraph say that sequencing info available for 122 index cases, but 2 paragraphs down state that 99 were alpha and 24 delta which would make for 123.
- Will the 72 individuals whose samples hadn’t been sequenced ever have this done? Would be interesting to see how well actual sequences match predictions based on circulation.
- Nitpicky, but I think saying that you failed to detect any difference between the vaccines would more appropriate than saying there was no difference.
- “At two to three days the effect in participants fully vaccinated with ChAdOx1 was slightly less pronounced.” Is this effect also a decline? Would be good to state clearly what this effect was.

Is the work clearly and accurately presented and does it cite the current literature?

Yes

If applicable, is the statistical analysis and its interpretation appropriate?

Yes

Are all the source data underlying the results available to ensure full reproducibility?

Yes

Is the study design appropriate and is the work technically sound?

Yes

Are the conclusions drawn adequately supported by the results?

Yes

Are sufficient details of methods and analysis provided to allow replication by others?

Partly

Reviewer Expertise:

Infectious disease epidemiology

I confirm that I have read this submission and believe that I have an appropriate level of expertise to confirm that it is of an acceptable scientific standard, however I have significant reservations, as outlined above.

# Response

**Published:** 2023-10-19

**Authors:** Stefan Flasche

**Affiliations:** LSHTM, UK

Thanks for the thorought review. We had sumbitted a new version that add clarifications as suggested. 

 In detail: 

- Why the difference in age groups for case and contact comparisons?
  -> included: “The different age grouping for cases and contacts were chosen because of the different age distribution in both groups (Table 2)”
- I’m not sure about the independence assumption between protective effect of vaccinated contacts and reduced transmission of vaccinated cases. Wouldn’t there be some clustering between similar groups?
  -> We believe the assumption of independence is reasonable: one is the protection against detectable infection in contacts and one is the protection against onward transmission in breakthrough infections (cases). We agree though that the formulation in the manuscript is potentially confusing and so we have changed this to “The model independently estimates both a protective effect for vaccinated contacts against infection and a reduction in transmission for vaccinated cases” to avoid suggesting that this would be a major assumption.
-  Should consider adding additional detail on complexity prior. It would also be helpful for a sentence or two on why precision is used here for those less familiar with its benefits for Bayesian modeling.
   -> we included: “We included penalised complexity priors to favour the NULL model of no protective vaccine effect against transmission.” We used precision for parameterisation as this is the standard parameterisation in the R package used.
- Would recommend changing by a factor of 2 to something more intuitive like doubling or 2x causes unnecessary confusion for folks who don’t frequently use this terminology.
  -> done
- Should clearly state which age group the case transmission prior is for.
   -> done
- Not clear where in the paper by Yousaf et al., the estimate comes from?
  -> we had written: “and from Yousaf *et al.*, ^18^ cited in Goldstein *et al.*, ^19^ for case transmission…” but changed to “similar to post hoc calculations in Goldstein et al based on Yousaf et al, for relative transmissibility of a >50y old case” to increase clarity that Yousaf et al did not calculate this themselves but that the value is based on a post-hoc analyses similar to that by Goldstein et al.
- Negative serial interval?
  -> negative serial intervals are possible in principle but unlikely and may point to misclassification instead. Hence we have included only household in which the assumption that the index case was indeed the first case in the household, resulted in serial intervals larger than -2 days.
-  90% of index cases tested positive at least once in the week prior to recruitment, what about the other 10%?
  -> It says in the manuscript: “Of the included individuals, 175 index cases (90%) and 113 (41%) contacts tested positive for SARS-CoV-2 at least once in the week SINCE recruitment”. The other 10% tested positive before recruitment but not in the swabs following enrolment.
- Based on how index cases were defined/identified they must have tested positive to be recruited, what range did you see in this group in days between positive test and recruitment?
  -> Afraid we did not store this information.
-  In one paragraph say that sequencing info available for 122 index cases, but 2 paragraphs down state that 99 were alpha and 24 delta which would make for 123.
  -> thanks for picking this up. This analysis was done in real time and thus for the main statistical analysis, that succeeded the phylogenetic analysis, we had marginally more sequencing information. We included some language to clarify this.
- Will the 72 individuals whose samples hadn’t been sequenced ever have this done? Would be interesting to see how well actual sequences match predictions based on circulation.
  -> we agree but only a subset of samples at the time have been sent for sequencing so I am afraid we will never know.
- Nitpicky, but I think saying that you failed to detect any difference between the vaccines would more appropriate than saying there was no difference.
  -> fair point. Changed to “indicate that at 95% credibility there is no evidence for a difference between the effectiveness of the two vaccine products”
-  “At two to three days the effect in participants fully vaccinated with ChAdOx1 was slightly less pronounced.” Is this effect also a decline? Would be good to state clearly what this effect was.
  -> changed to “In participants fully vaccinated with ChAdOx1 the effect was slightly less pronounced with PCR positivity declining two to three days earlier in vaccinees than in unvaccinated.”
